# Physician Perspectives on Ambient AI Scribes

**DOI:** 10.1001/jamanetworkopen.2025.1904

**Published:** 2025-03-24

**Authors:** Shreya J. Shah, Trevor Crowell, Yejin Jeong, Anna Devon-Sand, Margaret Smith, Betsy Yang, Stephen P. Ma, April S. Liang, Clarissa Delahaie, Caroline Hsia, Tait Shanafelt, Michael A. Pfeffer, Christopher Sharp, Steven Lin, Patricia Garcia

**Affiliations:** 1 Department of Medicine, Stanford University School of Medicine, Stanford, California; 2 Stanford Healthcare AI Applied Research Team, Division of Primary Care and Population Health, Stanford University School of Medicine, Stanford, California; 3 Geriatric Research Education and Clinical Center, Veterans Administration Healthcare System, Palo Alto, California; 4 Technology and Digital Solutions, Stanford Medicine, Stanford, California; 5 WellMD Center, Stanford University School of Medicine, Stanford, California

## Abstract

**Importance:**

Limited qualitative studies exist evaluating ambient artificial intelligence (AI) scribe tools. Such studies can provide deeper insights into ambient AI implementations by capturing lived experiences.

**Objective:**

To evaluate physician perspectives on ambient AI scribes.

**Design, Setting, and Participants:**

A qualitative study using semistructured interviews guided by the Reach, Efficacy, Adoption, Implementation, Maintenance/Practical, Robust Implementation, and Sustainability Model (RE-AIM/PRISM) framework, with thematic analysis using both inductive and deductive approaches. Physicians participating in an AI scribe pilot that included community and faculty practices, across primary care and ambulatory specialties, were invited to participate in interviews. This ambient AI scribe pilot at a health care organization in California was conducted from November 2023 to January 2024.

**Main Outcome and Measures:**

Facilitators and barriers to adoption, practical effectiveness, and suggestions for improvement to enhance sustainability.

**Results:**

Twenty-two semistructured interviews were conducted with AI pilot physicians from primary care (13 [59%]) and ambulatory specialties (9 [41%]), including physicians from community practices (12 [55%]) and faculty practices (10 [45%]). Facilitators to adoption included ease of use, ease of editing, and generally positive perspectives of tool quality. Physicians expressed positive sentiments about the impact of the ambient AI scribe tool on cognitive demand (16 of 16 comments [100%]), temporal demand (28 comments [62%]), work-life integration (10 of 11 comments [91%]), and overall workload (8 of 9 comments [89%]). Physician perspectives of the impact of the ambient AI scribe tool on their engagement with patients were mostly positive (38 of 56 comments [68%]). Barriers to adoption included limited functionality with non-English speaking patients and lack of access for physicians without a specific device. Physician perspectives on accuracy and style were largely negative, particularly regarding note length and editing requirements. Several specific suggestions for tool improvement were identified, and physicians were optimistic regarding the potential for long-term use of ambient AI scribes.

**Conclusion and Relevance:**

In this qualitative study, ambient AI scribes were found to positively impact physician workload, work-life integration, and patient engagement. Key facilitators and barriers to adoption were identified, along with specific suggestions for tool improvement. These findings suggest the potential for ambient AI scribes to reduce clinician burden, with user-centered recommendations offering practical guidance on ways to improve future iterations and improve adoption.

## Key Points

**Question:**

What are physician perspectives on the use of ambient AI scribes, including adoption, effectiveness, and opportunities for improvement in workflow integration?

**Findings:**

In this qualitative study of 22 physician interviews, ambient AI scribes were found to positively impact physician workload, work-life integration, and patient engagement. Key facilitators and barriers to adoption were identified, and physicians offered specific suggestions for tool improvement.

**Meaning:**

These findings suggest that there is potential for ambient AI scribes to reduce physician burden, with user-centered recommendations providing practical guidance on scaling this technology and addressing barriers to adoption.

## Teaser

This qualitative study evaluates physician perspectives about using ambient artificial intelligence (AI) scribes in clinical practice.

## Introduction

Clinical documentation burden has emerged as a major contributor to clinician burnout.^1,2,3,4,5,6,7,8,9,10,11,12^ A recent innovation to alleviate this burden is the use of ambient artificial intelligence (AI) scribes, particularly those leveraging generative AI driven by large language models (LLMs).^13,14^ Ambient AI scribes work by transcribing conversations between clinicians and patients in real time; these transcripts are then used to generate a structured draft clinical note for clinicians to review and edit. Early findings suggest ambient AI scribes can help to reduce clinician burden and burnout.^14,15,16^

Limited qualitative studies exist for the evaluation of ambient AI scribe tools.^17^ Such studies can offer deeper insights into ambient AI implementations by capturing lived experiences, identifying key facilitators and barriers to adoption, and elucidating the specific factors that make these tools beneficial. Qualitative evaluations can also provide crucial feedback for scaling these technologies across organizations, and offer user-centered recommendations for improvement, which can be used to enhance future iterations.^18,19,20^

This qualitative study aims to assess physician perspectives on an ambient AI scribe pilot through semistructured interviews, guided by the Reach, Efficacy, Adoption, Implementation, Maintenance/Practical, Robust Implementation, and Sustainability Model (RE-AIM/PRISM) framework.^20,21,22,23^ This framework is particularly well suited for evaluating the multifaceted impact of health technology implementations, capturing facilitators and barriers to adoption, practical effectiveness, and suggestions for improvement to enhance sustainability.

## Methods

The institutional review board (IRB) at Stanford University determined that this study met the criteria for quality improvement and was exempt from the need for IRB–mandated consent. The Standards for Reporting Qualitative Research (SRQR) reporting guideline for qualitative research were followed using the 21-item checklist.^24^

Purposive sampling was used to recruit a range of physicians who participated in a 3-month ambient AI scribe pilot^11,12^ with Nuance Dragon Ambient eXperience Copilot from November 2023 to January 2024 (n = 48), with varying levels of ambient AI scribe tool use (low, medium, and high). Additionally, physicians who voluntarily dropped out of the pilot were invited to participate (n = 2). All participants were directly contacted via email and invited to take part in an optional interview. Physicians in the pilot study were required to notify patients and others present in the room about being recorded prior to activating the ambient AI scribe tool during each visit.

The RE-AIM PRISM framework guided the evaluation design. A semistructured interview guide was developed to elicit open-ended responses from physicians. The interview guide remained largely unchanged, with only minor adjustments made to improve the flow of conversation after the first few participants. A comprehensive code book was developed to guide systematic analysis, rooted in the RE-AIM PRISM framework, and informed by prior work to evaluate clinician perspectives of AI-generated draft replies to patient inbox messages.^21^ The text of the interview guide is available in eMethods in Supplement 1, and the text of the code book is available in the eTable in Supplement 1.

### Data Analysis

Semistructured interviews were conducted virtually via Zoom between March and April 2024, and sessions were recorded with participants’ verbal consent. Because licenses were extended for all participating physicians, which allowed continued tool use beyond the initial 3-month pilot, most physicians were still using ambient AI scribes at the time of the interviews. Transcripts from the recordings were used for thematic analysis, which included both inductive and deductive approaches. Four qualitative researchers (T.C., Y.J., B.Y., and A.D.) independently coded phrases using the deductive codebook, achieving an interrater reliability of 76%. During this process, inductive codes were introduced to capture emerging themes. Code counts for each category were summarized first, followed by sentiment analysis. Phrases could be assigned multiple codes, each with a positive, negative, or neutral valence. All qualitative coding was conducted using Dedoose version 9.2.4 (Dedoose). The study was conducted from November 2023 to January 2024. Data management complied with our institutional policies, and all data for this study was stored on secure platforms appropriate for high-risk protected health information data.

## Results

Among 50 eligible physicians (48 who participated in the pilot and 2 who dropped out of the pilot), 22 completed the study with semistructured interviews about the AI pilot program (44%). Twelve physicians were from community practices (55%) and 10 were from faculty practices (45%). Thirteen physicians were from primary care specialties (59%) and 9 were from ambulatory specialties (41%) (Table 1). Among the interviewed physicians, 9 physicians (41%) were in the high-use group (ie, more than 70% use), 6 physicians (27%) were in the medium-use group (ie, 30% to 70% use), 5 physicians (23%) were in the low-use group (ie, less than 30% use), and 2 physicians (9%) dropped out of the pilot. Our analysis focused on 3 major aspects of the implementation: facilitators and barriers to adoption, practical effectiveness, and suggestions for improvement to enhance sustainability (Table 2).

**Table 1.** Characteristics of Interview Participants

Characteristic	Participants, No. (%)
Community practice	
Primary care^a^	8 (36)
Ambulatory specialty^b^	4 (18)
Faculty practice	
Primary care^a^	5 (23)
Ambulatory specialty^c^	5 (23)
Utilization rate	
High	9 (41)
Medium	6 (27)
Low	5 (23)
Drop out	2 (9)

^a^ Includes family medicine, internal medicine.

^b^ Includes otorhinolaryngology, gastroenterology, ophthalmology, orthopedic surgery.

^c^ Includes cardiology, immunology, rheumatology.

**Table 2.** Physician Interview Thematic Analysis and Representative Quotes

Theme	Representative quotations		No. of comments				Physician volume, No.
	Comment type	Quote	Positive	Neutral	Negative	Code count	
Temporal demand	Positive	“Overall, it did save time. Like those days where I tended to use [the AI scribe tool] more, it definitely saved me time. I would say in a 30 minute patient interaction, it saved me about 5 minutes, which is really huge.”	28 (62)	4 (9)	13 (29)	45	20
	Negative	“It’s probably actually taking me longer now because I’m doing more editing in the top part of my note than I would have done before.”					
Physician engagement with patient	Positive	“I feel like I can be more face-to-face with them and [have] more eye contact. I feel like I can establish a better relationship instead of staring at the computer and looking like I’m doing all my work and not paying attention to them.”	38 (68)	12 (21)	6 (11)	56	21
	Negative	“There have been just 2 patients who had initially consented and then they looked at the notes and then they felt uncomfortable and then they messaged me back and said, ‘don’t use this ever again.’”					
Note construction	Positive	“I mean, of course you got to edit some things. They’ll do some typos and, or they might say something or misunderstood something you said. It’s easy to correct.”	2 (4)	7 (16)	36 (80)	45	21
	Negative	“You have to go read it over and edit it, which just takes time.”					
Utility	Positive	“So it’s especially helpful for the focused 1 problem visits, the patients here for sore throat, for COVID, for a blood pressure check, things like that, where we can keep the patient focused and where it’s not surprising what the, the things that can be protocol, like very routine.”	61 (48)	12 (9)	54 (43)	127	22
	Negative	“I was very excited to start it, to start the, you know, I just was disappointed, you know, that, that it really wasn’t helping.”					
Ease of use	Positive	“And I find that that’s like pretty easy to do and doesn’t take a lot of mental effort to do. And it actually is more enjoyable.”	6 (55)	0	5 (45)	11	8
	Negative	“You couldn’t change the title, the way [the AI scribe tool] rolled out the product, and you could edit pieces of it, but if you try to move it or anything like that, you have to delete the entire section or copy it onto a clipboard.”					
Accessibility	Negative	“English as a second language is definitely a potential barrier, so I’m not using it if I have a translator that I’m working with for a patient.”	0	4 (19)	17 (81)	21	13
Quality	Positive	“In terms of, and I think this is a common theme we see, is that in terms of HPI, or history of present illness, [the AI scribe tool] does a fantastic job. Where it impressed me was the ability to understand, to delineate kind of social discussion with medically relevant discussion, and it did a pretty good job of getting the history in that portion.”	11 (58)	2 (11)	6 (32)	19	13
	Negative	“Yeah, I would say so far it’s doing, I think, a great job of storytelling, but probably not organizing and synthesizing things in a way that a clinician thinks. And so that to me is probably one of the biggest downsides.					
Accuracy	Positive	“In general, I was pretty impressed with overall fidelity of transcription.”	5 (15)	0	28 (85)	33	15
	Negative	“It makes mistakes and typos and hears things wrong, occasionally homonyms and other things and I have to fix it because it sounds funny.”					
Completeness	Positive	“So instead of writing all that down I just [talk] and have the computer dictate. It’s worked great so far.”	5 (24)	0	16 (76)	21	15
	Negative	“I mean, it was okay, but it would miss some of the subtleties that you can do, because how you say things is different.”					
Formatting	Positive	“The assessment and plan I’m pretty happy [with]... I summarize and it breaks it down into the proper bullet point.”	1 (10)	2 (20)	7 (70)	10	8
	Negative	“So there would be patients that I just knew it was going to end up being a massive block of text if I use the tool. And I would much rather go in with my own free text, like short form documentation.”					
Length/brevity	Positive	“So it’s very good on history, sometimes too much detail, but oftentimes pretty good.”	3 (17)	0	15 (83)	18	11
	Negative	“Because what it does is, in the history section, it’s too verbose.”					
Style	Positive	“But I appreciate that it’s now more formal. It sometimes goes a little bit too far on that side and I’m just, if it’s something I care about, I would adjust it and that’s not a problem.”	2 (12)	6 (35)	9 (53)	17	8
	Negative	“Also, I felt that it was a little bit cold, in that it never used the patient’s name, and always just use pronouns.”					
Cognitive demand	Positive	“I don’t know if it saved time, but it saved anxiety.”	16 (100)	0	0	16	10
Workload	Positive	“I think the, you know, even though [the AI scribe tool] doesn’t do anything to help with the in-basket directly, by saving the documentation burden after hours, it, it sort of indirectly makes that less painful. So yeah, I think I’ve heard that from others and felt that myself, but just sort of the overall workload is less burdensome.”	8 (89)	1 (11)	0	9	6
Work-life integration	Positive	“But for those like you that are already prioritizing that, this is going to help them get home at the end of the day and not be, not have to feel like it’s a trade-off of like your life versus the engagement with your patients.”	10 (91)	1 (9)	0	11	8
Workflow changes	Positive	“In terms of like impact to workflow, it certainly changed it around, because I think before there was like, I could try to get the note done quickly, much more concise, and simpler, but still done. And now it’s going back and reviewing the note, certainly more content, more involved, maybe better.”	9 (41)	4 (18)	9 (41)	22	12
	Negative	“I think it’s hard for people to change in what they already do... And mainly the rate limiting factor is that I always forget my phone, to bring my phone in the patient’s room.”					
Future use	Positive	“I would say everybody needs to try it. So, I have colleagues who hear I’m doing it. They’re like, I saw your notes. And then they’re like, how did you get this? I want to do this too.”	16 (84)	2 (11)	1 (5)	19	10

### Facilitators and Barriers to Adoption and Workflow Integration

#### Facilitators and Barriers to Adoption

Facilitators to adoption included ease of use (eg, “it’s very fast, and I like how it’s just a 1 click setup”), ease of editing (eg, “they’ll do some typos and/or they might say something or misunderstood something you said. It’s easy to correct”), and low cognitive lift (eg, “I find that that’s like pretty easy to do and doesn’t take a lot of mental effort to do. And it actually is more enjoyable”). Of the 21 comments in which physicians expressed challenges related to the tool’s linguistic and device accessibility, 0 were positive comments, 4 were neutral (19%), and 17 were negative (81%). Specific barriers to adoption included limited functionality with non-English speaking patients and lack of access for physicians without a specific device. In rare instances, patients were reluctant to have the ambient AI scribe tool used during a visit.

#### Quality, Accuracy, and Completeness

The 19 comments about physician perspectives of the quality of the ambient AI scribe tool were predominantly positive, with 11 positive comments (58%), 2 neutral comments (11%), and 6 negative comments (32%). One physician remarked “where it impressed me was the ability to understand, to delineate kind of social discussion with medically relevant discussion, and it did a pretty good job of getting the history in that portion.” One physician noted both positive and negative aspects of the tool commenting that it did a “great job of storytelling, but probably not organizing and synthesizing things in a way that a clinician thinks.” The 33 comments about accuracy were largely negative, with 5 positive comments (15%), 0 neutral, 28 negative comments (85%). For example, 1 physician observed, “it makes mistakes and typos and hears things wrong, occasionally homonyms and other things and I have to fix it occasionally.” Despite these concerns, a physician was “pretty impressed with overall fidelity of transcription.” Physicians also had negative opinions on completeness (5 of 21 positive comments [24%], 0 neutral comments, and 16 of 21 negative comments [76%]), as a physician mentioned “it would miss some of the subtleties that you can do, because how you say things is different.”

#### Note Construction, Style, and Use

The 45 comments regarding physician perspectives on note construction were predominantly negative (2 positive comments [4%], 7 neutral comments [16%], 36 negative comments [80%]), with 10 comments providing additional negative feedback on formatting (1 positive comment [10%], 2 neutral comments [20%], 7 negative comments [70%]). Additionally, 18 of the comments from the physicians provided multiple examples about length or brevity (3 positive comments [17%], 0 neutral comments [0%], 15 negative comments [83%]), describing the tool as “too verbose in the history section” and containing “too much detail [on history], but oftentimes pretty good.” One physician commented that during some visits, “I would much rather go in with my own free text, like short form documentation.” The 127 comments about utility were balanced (61 positive [48%], 12 neutral [9%], 54 negative [43%]); a physician mentioned “it’s especially helpful for the focused, 1-problem visits, the patients here for sore throat, for COVID, for a blood pressure check […] things that can be protocol, like very routine.”

### Practical Effectiveness

#### Cognitive Demand and Temporal Demand

Physician sentiments about the impact of the ambient AI scribe tool on cognitive demand were all positive (16 of 16 positive comments [100%]; 0 neutral comments; 0 negative comments), with 1 physician commenting “I don’t know if it saved time, but it saved anxiety.” The 45 comments about the temporal demand were also overwhelmingly positive (28 positive comments [62%]; 4 neutral comments [9%]; 13 negative comments [29%]). One physician noted, “those days where I tended to use [the AI scribe tool] more, it definitely saved me time. I would say in a 30-minute patient interaction, it saved me about five minutes, which is really huge.” Although a minority view, another physician reported the opposite, “it’s probably actually taking me longer now because I’m doing more editing in the top part of my note than I would have done before.”

#### Workload and Work-Life Integration

Similarly, there were also positive comments on workload (8 of 9 positive comments [89%]; 1 of 9 neutral comment [11%]; and 0 negative comments), with a physician commenting that “by saving the documentation burden after hours, it sort of indirectly makes that less painful. I think I’ve heard that from others and felt that myself, but just sort of the overall workload is less burdensome.” There were also positive physician perceptions about work-life integration (10 of 11 positive comments [91%]; 1 neutral comment [9%]; and 0 negative comments). One physician noted that “this is going to help them get home at the end of the day and not be, not have to feel like it’s a trade-off of like your life versus the engagement with your patients.”

#### Workflow Changes

The 22 comments addressing perspectives about workflow changes were balanced with 9 positive comments (41%), 4 neutral comments (18%), and 9 negative comments (41%). One physician noted “in terms of like impact to workflow, it certainly changed it around, because I think before […], I could try to get the note done quickly, much more concise, and simpler, but still done. And now it’s going back and reviewing the note, certainly more content, more involved, maybe better.”

#### Physician Engagement With Patients

Physicians also mentioned several positive comments about the impact of the ambient AI scribe tool on their engagement with their patients (38 of 56 positive comments [68%]; 12 of 56 neutral comments [21%]; 6 of 56 negative comments [11%]). One physician mentioned “I feel like I can be more face-to-face with them and more eye contact. I feel like I can establish a better relationship instead of staring at the computer and looking like I’m doing all my work and not paying attention to them.” Another physician noted 2 patients that were initially fine with use of the tool during the visit but “then they looked at the notes and then they felt uncomfortable and then they messaged me back and said, don’t use this ever again.”

### Suggestions for Improvement to Enhance Sustainability

Physician perspectives regarding future use of the ambient AI scribe tool were mostly positive (16 of 19 positive comments [84%]; 2 of 19 neutral comments [11%]; 1 of 19 negative comments [5%]). One physician commented “I would say everybody needs to try it. I have colleagues who hear I’m doing it. They’re like, I saw your notes. And then they’re like, how did you get this? I want to do this too.”

#### Feedback on Updates Prior to Interviews

Physicians noted helpful technological improvements to the ambient AI scribe tool that occurred after the pilot. Multiple physicians appreciated the change in generated drafts that prompted the language to be more formal and medically geared compared with a prior version (eg, “an upgrade that happened where it was less colloquial and more formal language, which I do appreciate).” There was also a change to reduce redundancy in listing diagnoses, as 1 physician highlighted “the recent upgrade, it’s better at not duplicating the same diagnoses over and over in the assessment plan.” Lastly, there was mention of an improvement in separating the note content into specific problems or concerns, a physician noted “it initially did poorly, but later started to do a little bit better about chunking the information into sort of things around a specific issue or problem.”

#### Suggestions Future Tool Improvement

Physicians also identified several specific suggestions for improvement that they hoped to see in future versions of the ambient AI scribe tool. This included a request for the ambient AI scribe tool to capture patients’ correct gender identity and preferred pronouns without the need for a physician to prime the tool (eg, “when I’m using [the AI scribe tool], I do a 1-liner before I go into the room just so it gets my pronouns right because otherwise it’s harder to change after the fact”). Another area for improvement was for the ambient AI scribe tool to better capture information during the examination, without “verbalizing the [examination] out loud. That feels a little bit awkward.” Another physician commented on the “opportunity for specific verbal cueing, meaning you say specific phrases and it does a certain thing […] if I said things in a certain way, would it write it this way versus another.” Lastly, several physicians mentioned a request for the tool to have enhanced personalization features (eg, “I felt like I needed to adjust items to be more in my own voice”).

## Discussion

This study presented a qualitative evaluation of ambient AI scribes focusing on facilitators and barriers to adoption, insights on practical effectiveness, and suggestions for tool improvement. The use of physician interviews in conjunction with an implementation science framework allowed for unique and richer insights compared with survey-based studies of clinician perspectives. Major themes included predominantly positive perspectives on the impact of ambient AI scribes on temporal demand and patient engagement, predominantly negative perspectives on note construction, and balanced perspectives about use.

Facilitators to adoption included ease of use, ease of editing, and generally positive physician perspectives on tool quality. Barriers to adoption included accessibility challenges, such as limited functionality with non-English speaking patients and lack of access for physicians without a compatible mobile device. Physician perspectives on accuracy and style were largely negative, particularly regarding the length of notes and the editing requirements associated with the ambient AI scribe tool. Despite these concerns, some physicians noted that the longer content might provide a more comprehensive capture of the encounter that otherwise might not have been documented.

Physicians expressed substantially positive sentiments regarding the impact of the ambient AI scribe tool on cognitive demand, temporal demand, work-life integration, and overall workload. Given that a primary objective of ambient AI scribe tools is to reduce clinician burden and burnout, these findings are promising and build on similar results from prior studies. Most physicians also felt the ambient AI scribe tool positively impacted their engagement with patients. However, there were rare instances of patient discomfort with tool use during visits, including 1 physician with 2 patients who requested the tool not be used again in future visits. Evaluating patient perspectives on the use of ambient AI scribe tools is an area of ongoing research. Our findings identified an equal number of positive and negative comments about workflow changes, which suggests that ongoing thoughtful workflow integration could further reduce clinician burnout.

Physician interviews identified several potential areas for improvement. Suggestions elicited during the pilot period, such as the need for medical terminology updates and a more granular assessment and plan, were implemented prior to the interviews, and physicians viewed these changes positively. Other suggestions for improvement were shared back with the technology developers and have informed the roadmap for future enhancements. These examples illustrate the importance of eliciting and using clinician perspectives to guide thoughtful redesign of novel health care technologies. Qualitative assessments using semistructured interviews as seen in this study can promote collaboration between clinicians and technology developers to prioritize enhancements and tailor features that will ultimately drive adoption. As AI scribe functionalities evolve to include features beyond note generation, this collaboration and understanding between software developers and clinicians will become even more important to maximize impact.

### Limitations

This study has limitations, which include sampling bias, as most interviewed physicians were high users of the ambient AI scribe tool, followed by medium and low users. Although efforts were made to evenly sample high, medium, and low users, more high users volunteered to be interviewed, which may have contributed to recruitment bias. Both factors could lead to an overrepresentation of positive comments. In addition, physicians had only been using the tool for 3 to 6 months at the time of the interviews, and there is likely a learning curve associated with initial use that may have affected the results. Future studies should reassess clinician perspectives after a more extended period of use. The interviews, analysis, and interpretation in this study primarily focused on the act of note creation rather than broader functions of clinical notes. Future research should investigate the impact of ambient AI scribe tools on medical synthesis, clinical communication, and clinical reasoning. Finally, this study evaluates perspectives on a single vendor solution, which was evolving during the course of the study, and may limit generalizability.

## Conclusions

In one of the first qualitative evaluations of physician perceptions of an ambient AI scribe technology implementation, key facilitators and barriers to physician adoption were identified, as were suggestions to support future scalability. Physicians expressed positive sentiments about the impact of ambient AI scribes on cognitive demand, time, work-life integration, and overall perception of workload. Given the epidemic of clinician burnout, improving physician perception is just as crucial as other measures like time and productivity. Physicians’ suggestions for improvement are key to driving future enhancements to promote adoption. Future qualitative research is needed to explore clinician perspectives after a more extended period of use and investigate patient perspectives on ambient AI scribe tools.

## Notes

Supplement 1.
eMethods
**eTable. **Codebook Guided By RE-AIM PRISM Framework

Supplement 2.
Data Sharing Statement
