# Supplementary material for: Physician Perspectives on Ambient AI Scribes
Source: JAMA Netw Open. 2025 Mar 24;8(3):e251904. doi: 10.1001/jamanetworkopen.2025.1904 (PMC11933996; doi:10.1001/jamanetworkopen.2025.1904)
Supplement: Supplement 1. — eMethods eTable. Codebook Guided By RE-AIM PRISM Framework [file jamanetwopen-e251904-s001.pdf]

Supplemental Online Content

Shah S, Crowell T, Jeong Y, et al. Physician perspectives on ambient AI scribes. *JAMA Netw Open*. 2025;8(3):e251904. doi:10.1001/jamanetworkopen.2025.1904

eMethods.

eTable. Codebook Guided By RE-AIM PRISM Framework

This supplemental material has been provided by the authors to give readers additional information about their work.

© 2025 Shah S et al. JAMA Network Open.

eMethods

Interview Guide

Hello and thank you for taking the time to meet with me today. My name is [***], and I am part of the team supporting the DAX Copilot implementation. Our goal is to understand the experiences of users like yourself to enhance the utility and effectiveness of DAX Copilot.

Before we begin, I'd like to ask for your permission to record this interview. Thank you! The recording will only be used to ensure accuracy when reviewing your responses and summarizing themes across interviews. All information shared during this interview will be anonymized before it is presented or shared in any form.

Today, I have a series of questions related to your use of DAX Copilot. We are interested in both the positive aspects and the challenges you've encountered while using DAX Copilot. Are we okay to proceed?

Questions:

1. What types of patients do you typically see in terms of demographics and some of the common health conditions you see?
2. Have you worked with human scribes before?
3. Describe your level of utilization of DAX.
4. Based on your experience with DAX Copilot in its current form, what types of visits work well with DAX Copilot? What types of visits do not work well with DAX Copilot?
    a. Probing: New vs old; Telemed vs in-person; Complex vs simple; Tangential patient vs non-tangential; English second language; family members
    b. When you decided not to use DAX, what were the main reason(s) why?
5. Did the DAX Copilot tool meet your expectations for the quality of a note written by a human clinician? Why or why not?
    c. Did you notice any recurring issues with the text generated by the tool?
    d. Did you attempt any strategies to “improve” the tool? What were the results?
6. How have your patients reacted to the use of DAX Copilot during visits? What are the most common questions or concerns they raise?
    a. How many say no?
7. How has the introduction of DAX Copilot changed your interactions with your patients?
8. Compared to your previous methods of documentation, in what ways did DAX Copilot save or add time to your workflows?

© 2025 Shah S et al. JAMA Network Open.

a. If interviewee indicates time saved > With the saved from using DAX Copilot, what activities or tasks are you now able to allocate more time to that you couldn't before?

9. How well did DAX integrate into your existing workflows?
10. In what ways would you modify the tool to enhance its usefulness?

Additional Questions if time:

11. Do you have concerns about integrating this tool into your documentation workflows? (e.g. legal, liability, etc.)
12. What would you say are the main factors that will influence your decision to use this tool in the long-term?
13. What would you say are the main factors that will influence your decision to recommend DAX Copilot to a colleague?
14. Would you describe yourself as more excited or apprehensive about AI and its increasing impact on patient care and clinical workflows?
15. What organizational, reimbursement, regulatory, or policy related factors influence your decision to use DAX in your practice?

© 2025 Shah S et al. JAMA Network Open.

eTable. Codebook Guided By RE-AIM PRISM Framework

Framework	Domain	Sub-domain	Definition
RE-AIM	Reach	Participation	Themes related to participation of specific specialties and roles in the pilot
RE-AIM	Reach	Accessibility	Absence or presence of a desired feature of the tool (e.g., can't use tool for non-English speaker)
RE-AIM	Reach	Patient Engagement	Themes related to impact of the tool on provider-patient relationship, communication or patient experience (e.g., facetime with patient)
RE-AIM	Effectiveness	Cognitive Demand	Cognitive/mental demand associated with the documentation task (thinking)
RE-AIM	Effectiveness	Temporal Demand	Temporal demand, i.e., the time pressure or time-related aspects of the documentation task
RE-AIM	Effectiveness	Workload	Themes related to tool's impact on documentation workload

© 2025 Shah S et al. JAMA Network Open.

RE-AIM	Effectiveness	Work-Life-Integration	Themes related to the tool's impact on work-life integration
PRISM	Intervention	Provider Perspective_ Quality	Perceptions of the quality of note (i.e., is the tool good at doing what it's supposed to do?)
PRISM	Intervention	Provider Perspective_ Accuracy	Themes related to the accuracy of AI-generated note
PRISM	Intervention	Provider Perspective_Completeness	Themes related to missing information
PRISM	Intervention	Provider Perspective_Utility	How useful or helpful tool is
PRISM	Intervention	Provider Perspective_Ease- of-Use	Themes related how easy it was to use a tool
PRISM	Intervention	Provider Perspective_Length/Brevity	Perceptions of the length/brevity of note

© 2025 Shah S et al. JAMA Network Open.

PRISM	Intervention	Provider Perspective_Style	Themes related to the provider's documentation style (e.g., use of problem-based charting or themes related to their language/voice/tone)
PRISM	Intervention	Provider Perspective_Formatting	Themes related to formatting (visual / style)
PRISM	Intervention	Provider Perspective_Note Construction	Themes related to note construction and editing requirements
PRISM	Intervention	Provider Perspective_Future Use	Themes related to desire to use in the future or hope for implementation
PRISM	Intervention	Provider Perspective_Tool Improvement	Themes related to the tool's improvement overtime
PRISM	Implementation	Implementation_Workflow changes	Impact of tool on workflows

© 2025 Shah S et al. JAMA Network Open.
