# Supplementary material for: Physician Perspectives on Ambient AI Scribes
Source: JAMA Netw Open. 2025 Mar 24;8(3):e251904. doi: 10.1001/jamanetworkopen.2025.1904 (PMC11933996; doi:10.1001/jamanetworkopen.2025.1904)
Supplement: Supplement 2. — Data Sharing Statement [file jamanetwopen-e251904-s002.pdf]

Data Sharing Statement

Shah. Physician Perspectives on Ambient AI Scribes. *JAMA Netw Open*. Published March 24, 2025. doi:10.1001/jamanetworkopen.2025.1904

Data
Data available: No
